# XELIRI compared with FOLFIRI as a second-line treatment in patients with metastatic colorectal cancer

**DOI:** 10.3892/ol.2014.2335

**Published:** 2014-07-10

**Authors:** CHENGXU CUI, CHANG SHU, YI YANG, JUNBAO LIU, SHUPING SHI, ZHUJUN SHAO, NAN WANG, TING YANG, SONGNIAN HU

**Affiliations:** 1 Department of Oncology, Cancer Institute and Hospital, Chinese Academy of Medical Sciences, Beijing 100021, P.R. China; 2 CAS Key Laboratory of Genome Sciences and Information, Beijing Institute of Genomics, Chinese Academy of Sciences, Beijing 100101, P.R. China; 3 Department of Oncology, Beijing Chaoyang San Huan Cancer Hospital, Beijing 100122, P.R. China

**Keywords:** FOLFIRI, XELIRI, UGT1A, polymorphism, haplotype

## Abstract

The aim of this study was to compare the efficacy, safety and survival rate of a treatment regimen comprising capecitabine plus irinotecan (XELIRI) to those of a standard regimen comprising leucovorin, fluorouracil and irinotecan (FOLFIRI), to determine the correlation among the inherited genetic variations in *UGT1A1*, *UGT1A7* and *UGT1A9*. A total of 84 consecutive patients with histologically confirmed metastatic colorectal cancer (mCRC) were included in the study. All patients were treated with FOLFIRI or XELIRI. The median progression-free survival time was 4.4 months for FOLFIRI and 5.7 months for XELIRI (hazard ratio=1.35; 95% confidence interval, 0.83–2.21; P=0.22). When compared with FOLFIRI (6.34%), XELIRI was associated with lower rates of severe toxicity (3.29) (P=0.026) and similar disease control rates (69.57% for FOLFIRI and 61.11% for XELIRI; P=0.49). In total, 17 single nucleotide polymorphisms were identified, five of which revealed an association with grade 3/4 neutropenia, including UGT1A7*4; however, UGT1A1*28 and UGT1A1*6, which have been previously reported, were not significant. Additionally, H2 haplotypes, which include UGT1A9*22, and H5 and H7 haplotypes, which include UGT1A7*2, UGT1A7*3 and UGT1A7*4, were associated with a higher risk of severe neutropenia. In conclusion, XELIRI is an effective treatment regimen with acceptable response rates and tolerability for mCRC patients as a second-line treatment. Furthermore, inherited genetic variations in *UGT1A1*, *UGT1A7* and *UGT1A9* are associated with grade 3/4 neutropenia.

## Introduction

Colorectal cancer (CRC) is the second leading cause of cancer-related mortality worldwide (1). In China, CRC accounted for 10.56 and 7.80% of the total cancer incidence and mortality, ranking third and fifth, respectively. The incidence and mortality rate of CRC has continued to increase steadily (2).

Fluorouracil (5-FU) has been the mainstay of treatment for CRC for almost half a century (3). As an oral fluoropyrimidine, capecitabine has been rationally designed to simulate infusional 5-FU (4). Various phase III trials have demonstrated that capecitabine is at least equivalent to 5-FU with respect to progression-free survival (PFS) and overall survival in the first-line treatment of metastatic CRC (mCRC), and exhibits a superior safety profile (5–7).

FOLFIRI, the combination of irinotecan plus fluoropyrimidines, leucovorin (LV) and 5-FU, is a standard second-line treatment option for advanced CRC (8–10). XELIRI is a combination of irinotecan plus capecitabine; therefore, XELIRI includes capecitabine in place of the 5-FU and LV used in the FOLFIRI regimen. As a result, XELIRI may simplify the treatment process and reduce the complications of the central venous catheter that is required for treatment with 5-FU in the FOLFIRI regimen (11–13). However, XELIRI is less commonly used, and is not recognized as a standard chemotherapy regimen. Furthermore, the clinical results available for the XELIRI regimen are limited.

Randomized controlled trials comparing XELIRI with FOLFIRI in the treatment of mCRC have revealed various results. In the BICC-C study, a significantly shorter PFS was noted for the XELIRI regimen, which was also associated with higher toxicity (10). By contrast, in a randomized prospective phase II trial, no significant differences were observed between PFS and toxicity for the XELIRI and FOLFIRI regimens (14). However, the genetic background of patients has been neglected in the majority of clinical trials and, therefore, it is possible that these conflicting results are associated with genetic variations.

Any administered drug may have a therapeutic effect in certain patients but be ineffective in others. Furthermore, certain patients may suffer adverse effects, where others are unaffected. Personalized medicine is tailored to provide an individualized treatment and to predict the clinical outcome of a variety of treatment regimens. The use of genetic information is one of the core elements in personalized medicine.

The interindividual variability, efficacy and toxicity of irinotecan, which is the common drug in the FOLFIRI and XELIRI regimens, has been attributed predominantly to inherited genetic variations in the *UGT1A* gene. *UGT1A1*, *UGT1A7* and *UGT1A9* are three significant members of the *UGT1A* subfamily; each isoform comprises a typical exon 1 and four identical downstream exons, and exon 1 is regulated by its own promoter (15). In 2005, the US Food and Drug Administration amended the direction of irinotecan, by appending additional toxicity and dosing warnings relating to the UGT1A1*28 allele (16). Whilst the frequency of UGT1A1*28 is low in East Asian populations, UGT1A1*6, which has only been identified in Asian populations, has been associated with toxicity in patients treated with irinotecan (17,18). *UGT1A7* and *UGT1A9* genotypes have also been reported to be predictors of response and toxicity in patients treated with irinotecan-based regimens (19). However, the toxicity and efficacy of irinotecan remains unpredictable.

The aim of the current study was to compare the efficacy, safety and survival rate of the XELIRI regimen to those of the standard FOLFIRI regimen. The functional regions of *UGT1A1*, *UGT1A7* and *UGT1A9* were sequenced and comprehensively analyzed for genetic polymorphisms, to determine the correlation between inherited genetic variations, and the efficacy and safety of these irinotecan-based regimens.

## Patients and methods

### Patients

Between 2009 and 2013 at the Cancer Institute and Hospital, Chinese Academy of Medical Sciences (Beijing, China), a total of 84 consecutive patients with histologically confirmed mCRC were included in the study. Each patient provided written informed consent. All patients were treated with FOLFIRI or XELIRI. The study was approved by the ethics committee of Beijing Chao-Yang Sanhuan Cancer Hospital (Beijing, China).

### Eligibility criteria

The following inclusion criteria were used in this study: Age, >18 years; Eastern Cooperative Oncology Group (ECOG) performance status (PS) of 0–2; adequate bone marrow, hepatic and renal function (absolute neutrophil count, >1,500/μl; hemoglobin levels, >9.0 g/dl; platelet count, >75,000/μl; total serum bilirubin levels, <1.5-fold the upper normal limit (UNL); alanine aminotransferase/aspartate aminotransferase ratio, <2.5-fold the UNL; and serum creatinine levels, <1.6 mg/dl or creatinine clearance, >40 ml/min).

### Exclusion criteria

The exclusion criteria included the following: Inadequately controlled hypertension; unstable angina pectoris; and history of myocardial infarction, stroke or transient ischemic attack, pulmonary embolism, or deep vein thrombosis within six months prior to treatment.

### Chemotherapy

The FOLFIRI or XELIRI regimen was administered until progressive disease (PD), unacceptable toxicity, patient refusal or a medical decision to discontinue treatment. The FOLFIRI regimen consisted of 180 mg/m^2^ irinotecan intravenously (IV) over 90 min, 400 mg/m^2^ LV IV over 2 h, and 400 mg/m^2^ 5-FU IV bolus, followed by 2,400 mg/m^2^ 5-FU IV over a 46-h infusion, all administered on day one, every two weeks. The XELIRI regimen consisted of 120 mg/m^2^ irinotecan IV on days one and eight, and 800 mg/m^2^ oral capecitabine twice per day on days one to 14, repeated every three weeks.

### Evaluation and statistical analysis

Baseline evaluations consisted of physical examination, complete medical history, electrocardiography, ECOG PS, a complete blood count, hepatic and renal function tests, and assessment of serum carcinoembryonic antigen levels. All patients received an abdominopelvic computed tomography (CT) or magnetic resonance imaging (MRI) scan, and chest X-ray or CT/MRI of the chest. During treatment, a follow-up CT/MRI of the abdomen and pelvis, and chest X-ray or chest CT/MRI were performed every six weeks. Assessments were performed every three courses until PD or upon the discontinuation of chemotherapy. Tumor response classification was based on the Response Evaluation Criteria in Solid Tumors guidelines (20), while toxicity was graded according to the National Cancer Institute Common Toxicity Criteria, version 3.0 (21).

The primary efficacy endpoint was PFS, which was defined as the time from initiation of treatment to the first documentation of PD, or to the date of mortality or loss to follow-up. The secondary efficacy endpoint included overall response (complete + partial response) and toxicity.

For the comparison of the two chemotherapy regimens, Fisher’s exact test and Student’s t-test were used. Kaplan-Meier method was used for PFS analysis. The hazard ratio (HR) and 95% confidence interval (CI) for the treatment comparisons were obtained from a Cox proportional hazards model. All statistical tests were two-sided, and P<0.05 was considered to indicate a statistically significant difference between the two treatment regimens. All results were analyzed using IBM SPSS 19.0 software (IBM, Armonk, NY, USA).

### Genotyping and genetic analysis

Blood samples were obtained from 22 mCRC patients (10 placed into the FOLFIRI group and 12 into the XELIRI group) for isolation of genomic DNA at least one week prior to commencing chemotherapy. Genomic DNA was isolated from peripheral blood samples using the QIAamp DNA blood mini kit (Qiagen, Valencia, CA, USA). To screen the single nucleotide polymorphisms (SNPs) in *UGT1A1*, *UGT1A7* and *UGT1A9*, the gene regions were sequenced, including its promoter and exon 1, using the DYEnamic ET terminator cycle sequencing kit (GE Healthcare, Chalfont St. Giles, UK) on the ABI Prism 3730xl DNA analyzer (Applied Biosystems, Foster City, CA, USA). Following pre-denaturation at 93°C for 3 min, amplification was performed under the following conditions for 32 cycles: Denaturation at 95°C for 30 sec; annealing at 58°C for 40 sec; and extension at 72°C for 2 min. The primer sequences used are shown in Table I.

All sequences were analyzed with Phred, Phrap, Consed and Polyphred programs (University of California, Oakland, CA, USA; http://elcapitan.ucsd.edu/hyper/polyphred.usage.html) and were compared with the reference sequence NC_000002.11 to evaluate genetic variations. Estimating allele frequencies, testing the Hardy-Weinberg equilibrium, measuring pairwise linkage disequilibrium (LD) and estimating haplotype frequency were performed using Haploview 4.2 (Broad Institute of MIT and Harvard, Cambridge, MA, USA; http://www.broad.mit.edu/mpg/haploview/). Correlations between the SNPs or haplotypes and toxicity or response were analyzed by Pearson’s χ^2^ test. On account of the exploratory nature of this study, no adjustments were made for multiple comparisons.

## Results

### Patient characteristics

Between 2009 and 2013 at the Cancer Institute and Hospital, Chinese Academy of Medical Sciences, a total of 84 consecutive patients with histologically confirmed mCRC were included in this study. In total, 46 patients were treated with the FOLFIRI regimen and 38 patients received the XELIRI regimen. The patient baseline characteristics of the two chemotherapy regimens are summarized in Table II. No statistically significant differences were observed between the baseline characteristics for the two regimens.

### Efficacy

**Figure 1:**
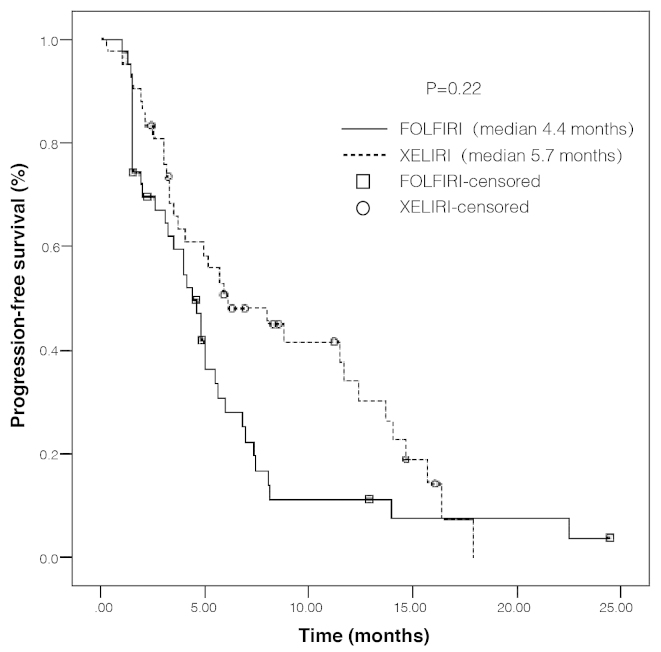
Progression-free survival for patients treated with FOLFIRI or XELIRI regimens. FOLFIRI, leucovorin, fluorouracil and irinotecan; XELIRI, capecitabine and irinotecan

The efficacy of the treatment groups is shown in Table III. The disease control rates did not differ significantly between the two chemotherapy arms (69.57% for FOLFIRI and 61.11% for XELIRI; P=0.49). Although the overall response rate of the FOLFIRI group was markedly higher than that of the XELIRI group (21.74% for FOLFIRI and 13.89% for XELIRI), the differences in the overall response rate between the two groups did not appear to be statistically significant (P=0.40). The PFS of patients in the FOLFIRI and XELIRI groups is presented in Fig. 1. The median PFS time for the patients in the FOLFIRI group was 4.4 months, and 5.7 months in the XELIRI group. Although PFS improved for patients who received XELIRI when compared with FOLFIRI, no statistically significant differences were observed in PFS between the two groups (HR=1.35 for disease progression or mortality; 95% CI, 0.83–2.21; P=0.22).

### Tolerability

Adverse events of any grade in the FOLFIRI and XELIRI treatment groups are shown in Table IV. The most common grade 3/4 adverse events associated with FOLFIRI and XELIRI were neutropenia (26.09% in the FOLFIRI group and 5.26% in the XELIRI group) and leukopenia (17.39% in the FOLFIRI groups and 10.53% in the XELIRI group). FOLFIRI was associated with higher rates of grade 3/4 leukopenia, neutropenia, thrombocytopenia, nausea and vomiting. However, these differences were not statistically significant, with the exception of neutropenia, which was the most frequently reported grade 3/4 hematological toxicity (P=0.03). Hand-foot syndrome is the most common adverse event associated with capecitabine; however, no hand-foot syndrome of grade 3/4 occurred in either of these two groups.

### Inherited genetic variations in UGT1A gene

In total, 17 SNPs were identified across the sequencing regions of the *UGT1A1*, *UGT1A7* and *UGT1A9* genes; all of the SNPs under investigation were in Hardy-Weinberg equilibrium (P>0.05). However, no significant correlation was observed for efficacy (Table V). Certain SNPs exhibited an association with severe toxicity; however, UGT1A1*28 and UGT1A1*6, which have been previously reported (17,18), were not significant (Table VI).

Among the detected variants, only the common SNPs with a minor allele frequency (MAF) of >10% were tested for pairwise LD. Two main linkage blocks were observed across the sequenced region, while two major haplotypes were identified by haplotype analysis using Haploview; ATA in block one (56.4%) and TCTCGT in block two (54.5%). Haplotype H7, which includes UGT1A7*2, UGT1A7*3 and UGT1A7*4, was found to correlate with the disease control rate (P=0.045) (Table VII); while haplotypes H2 and H5 were observed to correlate with a higher risk of severe neutropenia (Table VIII).

## Discussion

A number of phase II studies on XELIRI have suggested acceptable response rates and tolerability (22,23). However, in the phase III study by Fuchs *et al* (10), a significantly shorter PFS was noted for the XELIRI regimen, which was also associated with higher rates of severe vomiting, diarrhea and dehydration.

The present study investigated the second-line treatment of mCRC, and demonstrated that the XELIRI regimen, which is composed of irinotecan with oral capecitabine, offers similar disease control rates (69.57% for FOLFIRI and 61.11% for XELIRI; P=0.49) and longer PFS (median, 4.4 months for FOLFIRI and 5.7 months for XELIRI) when compared with FOLFIRI. Additionally, grade 3/4 leukopenia, neutropenia, thrombocytopenia, nausea and vomiting were less frequently observed in patients treated with the XELIRI regimen when compared with the FOLFIRI regimen; however, these were not significant, with the exception of neutropenia (P=0.03). Furthermore, no occurrence of hand-foot syndrome of grade 3/4 was observed in the XELIRI treatment group, which is the most common adverse event associated with capecitabine.

Taking the physical health of the patients into account, the dose of oral capecitabine was reduced to 800 mg/m^2^ twice a day on days 1–14. The lower overall response rate (21.74% for FOLFIRI and 13.89% for XELIRI; P=0.40) and reduced toxicity may result from the lower doses of the combination of capecitabine and irinotecan. Alternatively, another reason for this may be that the patients neglect one or more doses of the regular dosing schedule, as capecitabine is an oral drug that must be self-taken by the patient at home.

In conclusion, the present study demonstrated that XELIRI is an effective treatment regimen with acceptable response rates and tolerability for mCRC patients as a second-line treatment in addition to FOLFIRI.

Irinotecan, which is the common drug in FOLFIRI and XELIRI regimens, has a narrow therapeutic range, and severe toxicity may limit the dose that can be safely administered (24). The increasing knowledge of human genetic variations is likely to aid with personalized treatment.

The current study is different from previous studies, which have concentrated more on several specific alleles, including UGT1A1*28, UGT1A1*6, UGT1A7*2 and UGT1A9*22 (17,18,25,26). In order to obtain further information, the promoter and exon 1 of *UGT1A1*, *UGT1A7* and *UGT1A9* were screened, and 17 SNPs as well as two main linkage blocks were identified. When only the SNPs and haplotypes with MAF of >10% were considered to minimize the statistical discrepancy, no significant correlation with treatment efficacy was observed. In total, five SNPs were identified to reveal a correlation with grade 3/4 neutropenia, including UGT1A7*4; however, the correlation with UGT1A1*28 and UGT1A1*6, which has been repeatedly reported, was not significant. Furthermore, the H2 haplotype, which includes UGT1A9*22, and the H5 and H7 haplotypes, which include UGT1A7*2, UGT1A7*3 and UGT1A7*4, were associated with an increased risk of severe neutropenia. The limitations of this study are the exploratory nature and the limited sample size. Therefore, the results must be confirmed by additional studies comprising a larger number of patients and a more comprehensive assessment of variations in *UGT1A* in the future.

## Figures and Tables

**Table I:** Primer sequences.

Genes	Primer ID	Primer sequences
UGT1A1	U1_E1AF	TCGTCCTTCTTCCTCTCTGG
	U1_E1AR	GCAGTGCATGCAAGAAGAAT
	U1_E1BF	TGTCTGGCTGTTCCCACTTA
	U1_E1BR	CCAGAAGATGATGCCAAAGA
	U1_PF	GGTCATTCTCTACCCCAGCA
	U1_PR	AAAGCTGTCAGTCCACAAAGG
UGT1A7	U7_E1AF	AAACTCATATTGCAGCACAGG
	U7_E1AR	AAGTCAAAAATACCATTGGATGAA
	U7_E1BF	GGAAGATCACTGAATTGCACAG
	U7_E1BR	TTCCTCTGGGGGTAGTGTAGAA
	U7_PF	TCTTTCCGTCGAACATGAGA
	U7_PR	CACATTCACTGCCAATGATTTA
UGT1A9	U9_E1AF	CCAAGGCAAAGACCATAAGCTA
	U9_E1AR	CAAACTCCTGCAATTTGAAAAA
	U9_E1BF	CATATACCCTGGAGGATCTGGA
	U9_E1BR	CTGACGAGTACACGCATTGG
	U9_PF	CCTCTGACCTCAAGGAGTGC
	U9_PR	CAATGATTTACCCAAAAGAACAAG

F, forward; R, reverse.

**Table II:** Baseline patient characteristics.

	FOLFIRI (n=46)		XELIRI (n=38)		
			
Characteristics	n	%	n	%	P-value
Age, years					0.49
Median	54		53		
Range	29–77		30–72		
BSA, m^2^					0.21
Median	1.78		1.73		
Range	1.42–2.10		1.40–2.00		
Gender					0.17
Male	33	71.74	21	55.26	
Female	13	28.26	17	44.74	
Primary tumor site					0.66
Colon	28	60.87	25	65.79	
Rectum/rectosigmoid	18	39.13	13	34.21	
Metastatic sites					0.41
1	18	39.13	11	28.95	
2	21	45.65	17	44.74	
≥3	7	15.22	10	26.32	
ECOG PS					0.33
0	12	26.09	11	28.95	
1	34	73.91	25	65.79	
2	0	0.00	2	5.26	
TNM stage					0.27
IIIB	4	8.70	1	2.63	
IIIC	0	0.00	1	2.63	
IV	42	91.30	36	94.74	

FOLFIRI, leucovorin, fluorouracil and irinotecan; XELIRI, capecitabine and irinotecan; BSA, body surface area; ECOG PS, Eastern Cooperative Oncology Group performance status; TNM, tumor node metastasis.

**Table III:** Responses to treatment.

	FOLFIRI (n=46)		XELIRI (n=38)		
			
Response	n	%	n	%	P-value
CR	0	0.00	0	0.00	0.59^a^
PR	10	21.74	5	13.89	
SD	22	47.83	17	47.22	
PD	14	30.43	14	38.89	
Not assessable	0	0.00	2	5.56	
Overall response^a^	10	21.74	5	13.89	0.40
Disease control^b^	32	69.57	22	61.11	0.49

a Difference among the whole distribution of CR/PR/SD/PD;

b CR + PR;

c CR + PR + SD.

FOLFIRI, leucovorin, fluorouracil and irinotecan; XELIRI, capecitabine and irinotecan; CR, complete response; PR, partial response; SD, stable disease; PD, progressive disease.

**Table IV:** Drug-related adverse events.

	FOLFIRI (n=46)				XELIRI (n=38)				
			
	Grade 1/2		Grade 3/4		Grade 1/2		Grade 3/4		
					
Response	n	%	n	%	n	%	n	%	P-value
Hematological events									
Anemia	20	43.48	1	2.17	19	50.00	2	5.26	0.58
Leukopenia	22	47.83	8	17.39	18	47.37	4	10.53	0.66
Neutropenia	14	30.43	12	26.09	18	47.37	2	5.26	**0.03**
Thrombocytopenia	9	19.57	2	4.35	9	23.68	0	0.00	0.59
Non-hematological events									
Asthenia	1	2.17	0	0.00	3	7.89	0	0.00	0.32
Nausea	39	84.78	5	10.87	33	86.84	2	5.26	0.58
Vomiting	28	60.87	5	10.87	27	71.05	2	5.26	0.54
Mucositis	1	2.17	0	0.00	1	2.63	0	0.00	1.00
Diarrhea	10	21.74	2	4.35	13	34.21	2	5.26	0.39
Neurotoxicity	2	4.35	0	0.00	3	7.89	0	0.00	0.65
Hand-foot syndrome	0	0.00	0	0.00	1	2.63	0	0.00	0.45
Allergies	0	0.00	0	0.00	1	2.63	1	2.63	0.20

Bold P-value indicates significance (P<0.05). FOLFIRI, leucovorin, fluorouracil and irinotecan; XELIRI, capecitabine and irinotecan.

**Table V:** Association of UGT1A polymorphisms with efficacy.

				P-value^b^	
				
Genes	SNP ID	Allele^a^	MAF	Overall response	Disease control
*UGTIA9*	rs3806598		0.295	0.605	0.605
	rs45440791		0.023	0.613	0.613
	rs59870334	UGTIA9*22	0.429	0.767	0.670
*UGTIA7*	rs4530361		0.295	0.605	0.605
	rs28946877		0.167	0.168	0.949
	rs7586110		0.310	0.685	0.290
	rs7577677		0.295	0.605	0.605
	rs17868323	UGTIA7*2 and *3	0.432	0.749	0.749
	rs66534818	UGTIA7*2 and *3	0.432	0.749	0.749
	rs17868324	UGTIA7*2 and *3	0.432	0.749	0.749
	rs11692021	UGTIA7*3 and *4	0.295	0.605	0.605
	rs45462096		0.023	0.000	0.000
	rs17864686		0.159	0.184	0.825
*UGTIA1*	rs887829		0.091	0.368	0.548
	rs873478		0.045	0.468	0.468
	rs34815109	UGTIA1*28	0.114	0.292	0.792
	rs4148323	UGTIA1*6	0.250	0.361	0.068

a SNP ID not available for all alleles;

b Pearson’s χ^2^ test.

SNP, single nucleotide polymorphism; MAF, minor allele frequency.

**Table VI:** Correlation between UGT1A polymorphisms and severe toxicity.

				P-value^b^								
				
Genes	SNP ID	Allele^a^	MAF	Anemia	Neutropenia	Leukopenia	Thrombo-cytopenia	Nausea	Vomiting	Diarrhea	Allergies	Severetoxicity
*UGTIA9*	rs3806598		0.295	0.349	**0.032**	0.237	0.516	0.516	0.516	0.347	0.516	0.119
	rs45440791		0.023	0.825	0.688	0.688	0.825	0.825	0.825	0.749	0.825	0.445
	rs59870334	UGTIA9*22	0.429	0.834	0.203	0.203	0.834	0.000	0.000	0.762	0.834	0.186
*UGTIA7*	rs4530361		0.295	0.349	**0.032**	0.237	0.516	0.516	0.516	0.347	0.516	0.119
	rs28946877		0.167	0.195	0.237	1.000	0.517	0.000	0.000	0.347	0.517	0.770
	rs7586110		0.310	0.332	**0.041**	0.276	0.551	0.000	0.000	0.386	0.551	0.238
	rs7577677		0.295	0.349	**0.032**	0.237	0.516	0.349	0.349	0.347	0.516	0.382
	rs17868323	UGTIA7*2 and *3	0.432	0.842	0.211	0.211	0.842	0.207	0.207	0.773	0.842	0.490
	rs66534818	UGTIA7*2 and*3	0.432	0.842	0.211	0.211	0.842	0.207	0.207	0.773	0.842	0.490
	rs17868324	UGTIA7*2 and*3	0.432	0.842	0.211	0.211	0.842	0.207	0.207	0.773	0.842	0.490
	rs11692021	UGTIA7*3 and*4	0.295	0.349	**0.032**	0.237	0.516	0.516	0.516	0.347	0.516	0.119
	rs45462096		0.023	0.825	0.688	**0.011**	0.825	0.825	0.825	0.749	0.825	0.181
	rs17864686		0.159	0.177	0.252	0.957	0.529	0.529	0.529	0.362	0.529	0.640
*UGTIA1*	rs887829		0.091	0.647	0.405	0.487	0.647	0.647	0.647	0.507	0.647	0.620
	rs873478		0.045	**0.002**	0.565	0.565	0.752	0.752	0.752	0.647	0.752	0.682
	rs34815109	UGTIA1*28	0.114	0.604	0.660	0.660	0.604	0.604	0.604	0.453	0.604	0.858
	rs4148323	UGTIA1*6	0.250	0.403	0.128	0.612	0.403	0.403	0.403	1.000	0.403	0.469

a SNP ID not available for all alleles;

b Pearson’s χ^2^ test. Bold P-value indicates significance (P<0.05).

SNP, single nucleotide polymorphism; MAF, minor allele frequency.

**Table VII:** Correlation between UGT1A haplotypes and efficacy

				P-value^a^	
				
Block	ID	Haplotype	MAF	Overall response	Disease control
1	H1	ATA	0.564	0.725	0.544
	H2	CAG	0.295	0.605	0.605
	H3	AAA	0.141	0.222	0.851
2	H4	TCTCGT	0.545	0.633	0.753
	H5	GAGAAC	0.272	0.478	0.862
	H6	TCGAAT	0.136	0.232	0.232
	H7	GAGAAT	0.023	0.614	**0.045**
	H8	TCTCGC	0.015	0.692	0.118

a Pearson’s χ^2^ test. Bold P-value indicates significance (P<0.05).

MAF, minor allele frequency.

**Table VIII:** Correlation between UGT1A haplotypes and severe toxicity.

				P-value^a^								
				
Block	ID	Haplotype	MAF	Anemia	Neutropenia	Leukopenia	Thrombo- cytopenia	Nausea	Vomiting	Diarrhea	Allergies	Severe toxicity
1	H1	ATA	0.564	0.853	0.221	0.221	0.853	0.633	0.633	0.788	0.853	0.161
	H2	CAG	0.295	0.349	**0.032**	0.237	0.516	0.516	0.516	0.347	0.516	0.119
	H3	AAA	0.141	0.135	0.286	0.845	0.558	0.865	0.865	0.396	0.558	0.961
2	H4	TCTCGT	0.545	0.896	0.261	0.262	0.893	0.896	0.896	0.846	0.893	0.276
	H5	GAGAAC	0.272	0.376	**0.020**	0.178	0.461	0.376	0.376	0.286	0.461	0.250
	H6	TCGAAT	0.136	0.125	0.295	0.816	0.565	0.565	0.565	0.405	0.565	0.868
	H7	GAGAAT	0.023	0.824	0.695	0.690	0.832	0.824	0.824	0.759	0.832	0.452
	H8	TCTCGC	0.015	0.860	0.761	0.754	0.871	**0.000**	**0.000**	0.814	0.871	0.296

a Pearson’s χ^2^ test. Bold P-value indicates significance (P<0.05).

MAF, minor allele frequency.
